# A Pilot Feasibility Study of an Online Youth Tobacco Survey Administration among High School Students

**DOI:** 10.3390/ijerph19169911

**Published:** 2022-08-11

**Authors:** Kaitlyn M. Mazzilli, Michelle T. Bover Manderski, Cristine D. Delnevo, Mary Hrywna

**Affiliations:** 1 Center for Tobacco Studies, Rutgers Biomedical and Health Sciences, Rutgers University, New Brunswick, NJ 08901, USA; 2 Department of Biostatistics and Epidemiology, Rutgers School of Public Health, Rutgers University, Piscataway, NJ 08854, USA; 3 Department of Health Behavior, Society, and Policy, Rutgers School of Public Health, Rutgers University, Piscataway, NJ 08854, USA

**Keywords:** youth, adolescents, tobacco use, electronic cigarettes, survey feasibility

## Abstract

The COVID-19 pandemic restrictions forced many schools to shift to remote or hybrid learning, disrupting surveillance systems such as the New Jersey Youth Tobacco Survey, traditionally administered in schools by paper and pencil. In spring 2021, we conducted a feasibility study among a convenience sample of six public high schools to assess the use of an online survey to allow for remote participation. In each school, 4 to 6 classes were selected randomly, and all students within a sampled class were selected to participate in the survey. A total of 702 students completed surveys. School contacts were asked to provide qualitative feedback about the survey administration. Feedback was generally positive, with a few suggestions for improvement. Approximately 19% of students reported the ever use of e-cigarettes. Among current e-cigarette users, there was a shift in popularity from prefilled or refillable pods or cartridges (23.3%) to disposable e-cigarettes (53.5%). Less than 10% of current e-cigarette users reported using tobacco-flavored e-cigarettes, despite a statewide flavor ban on all other flavors.

## 1. Introduction

Tobacco use remains a leading cause of death in the United States, and tobacco use among youth and young adults in any form is considered unsafe [1]. Since 2014, the most commonly used tobacco product among U.S. youths was e-cigarettes [2,3,4]. In 2021, an estimated 2.06 million youths had used e-cigarettes within the past 30 days [4]. Most e-cigarettes contain nicotine, among other harmful substances [1]. Nicotine can harm adolescent brain development, impacting attention, learning, and memory [1]. Addiction to nicotine can lead to the regular use of tobacco products, resulting in long-term exposure to toxic chemicals [5]. Close to 90% of smokers start by the age of 18, highlighting the importance of supporting youth with relevant prevention and cessation efforts [1].

Since 1999, New Jersey has conducted biennial surveillance of tobacco use among New Jersey youth. The New Jersey Youth Tobacco Survey (NJYTS) is an adaptation of the National Youth Tobacco Survey (NYTS) developed by the Centers for Disease Control and Prevention (CDC), consisting of both CDC-recommended core questions and additional questions specific to New Jersey. In the previous iteration, the 2018 NJYTS found that 21.7% of students had used at least one tobacco product in the 30 days preceding that survey [6].

Previous administrations of the NJYTS took place in New Jersey public middle and high schools using a paper-and-pencil survey. Because of the emergency protocols that schools began to follow due to the COVID-19 pandemic, survey data collection could not take place in person and NJYTS could not be conducted as planned in fall 2020. As a result of the ongoing effects of the COVID-19 pandemic during 2021, schools were operating with some students attending in person, some attending remotely (virtually), and some attending in combinations of both (hybrid). In 2019, the National Youth Tobacco Survey was fully administered using an electronic survey version following an electronic pilot survey conducted in 2018, which found that an electronic survey was feasible, was well-accepted by respondents, and improved efficiency [7,8]. Thus, when COVID-19 disrupted typical school survey procedures, it prompted us to consider transitioning the NJYTS from a paper-and-pencil administration to an electronic survey administration. The 2021 NJYTS was conducted as a feasibility study to evaluate the process of administering a web-based student survey in a convenience sample of six public high schools to prepare to implement a web-based NJYTS in the fall of 2022. The purpose of this study was to assess a reasonable alternative to the previous paper-and-pencil in-person New Jersey Youth Tobacco Survey for future administrations, as well as to present preliminary data regarding tobacco use behavior, knowledge, and attitudes among a small convenience sample of New Jersey high school students. In addition to assessing school contacts’ perceptions about electronic survey procedures, the study also presents the latest data on youth tobacco use in New Jersey since 2018.

## 2. Materials and Methods

NJYTS typically employs a two-stage cluster design to obtain a representative sample of students in grades 9–12. The first stage of sampling includes a sampling frame that consists of all public high schools enrolling students in grades 9–12. However, for this 2021 feasibility study, our convenience sample consisted of six public high schools of varying enrollment sizes and economic diversity. Two schools were selected with enrollments of less than 1000 students; two schools with enrollments between 1000 and 2000 students; and two schools with enrollments of 2000 or more students. Each school that participated in the 2021 NJYTS received USD 500 for their participation. 

Consistent with NJYTS sampling procedures, the second stage of sampling involved selecting classrooms within each school using a sampling frame that consisted of an exhaustive list of classes that either met during a particular period of the day (e.g., 2nd period) or across an academic subject (e.g., English) required of all students. The sampling design ensured that all students had the same probability of selection and that all students had one, and only one, chance of being selected. To accomplish this, all classes were selected using a randomizer. In each participating school, four to six classes were selected at random, and all students within a sampled class were selected to participate in the survey. Data collection occurred between 10 May 2021 and 11 June 2021. A total of 792 students were randomly selected to participate and 702 students completed surveys, for a student response rate of 88.6%.

All six schools reported that every student was given access to an electronic device, so web-based survey access was possible at every school. Survey materials were designed with all three instructional models (in-person, virtual, or hybrid) in mind to support the administration of the web survey. Teachers were asked to read an introductory script and direct students to the online survey link. For those taking the survey remotely, teachers provided students with a unique five-digit classroom access code to log in to the survey. For those in person, teachers distributed sign-in cards with a unique five-digit access code to log in to the survey securely. Students in both situations were routed to a page displaying instructions and their rights as participants, and then were directed to the start of the survey. The survey administration procedures were designed to ensure that data were recorded in an anonymous fashion; no student names or identifiers were collected.

While students were taking the survey, teachers were asked to log in to a separate website and enter the same five-digit classroom access code in order to complete a form documenting class enrollment, returned permission forms, and student absences.

Once survey administration was completed, school contacts were asked to respond to several open-ended questions about the survey procedures. Questions focused on instructional clarity and the reporting of any issues relating to preparation for data collection, the process of data collection, and the completion of the survey.

The 2021 NJYTS included questions about ever use (i.e., any use, even one time) and current use (i.e., use on one or more of the previous 30 days) of cigarettes, cigars, smokeless tobacco (SLT), hookah, snus, electronic cigarettes (e-cigarettes), heated tobacco products, and nicotine pouches, as well as questions that assessed susceptibility to tobacco use, exposure to secondhand smoke, access to tobacco, and product use during the COVID-19 pandemic.

Sample demographics are reported with 95% confidence intervals (CI) to compare distributions among participating students with state enrollment levels. Frequencies and percentages of ever and current use of each tobacco product are reported, as well as e-cigarette use characteristics and perceived change in tobacco product use during the COVID-19 pandemic. All analyses were performed using SAS software version 9.4 (SAS Institute, Cary, NC, USA).

## 3. Results

### 3.1. Qualitative Data

Feedback from school contacts was generally positive. Contacts reported that the directions for administering the survey were clearly communicated, and the parental notification process was transparent. Teachers had minimal issues completing the enrollment information and reported that they received the technical support they needed. However, there were a few reported issues. One school reported that the links did not work properly because they were blocked by the school’s firewall and suggested that future links should be approved by school IT departments in advance. One teacher distributed permission forms and administered the survey to students in two classes as opposed to the one class that was selected. There was a suggestion to reduce the number of questions in the survey, and extra time was requested for students who required testing accommodations.

### 3.2. Survey Data

The demographics of the sample compared with New Jersey high school enrollments are presented in Table 1. This sample largely comprised Hispanic (37.6%) and Asian (25.4%) students. These groups were overrepresented in our sample when compared to New Jersey high school enrollments (28.7% and 10.5%, respectively). White (21.2%) and Black (8.3%) students were underrepresented in our sample when compared to New Jersey high school enrollments (44.6% and 14.1%, respectively). 

The prevalence of each product type is presented overall and by gender, race/ethnicity, and grade level in Table 2. The most common tobacco products ever tried were e-cigarettes (18.9%), followed by hookah (7.1%) and cigarettes (6.1%). The prevalence of having tried a cigarette was highest among 12th graders (13.2%).

Current use prevalence for each product is presented overall and by gender, race/ethnicity, and grade level in Table 3. The product used most frequently in the past 30 days was e-cigarettes (6.1%), followed by hookah (1.4%).

E-cigarette use characteristics among students who report current use are presented in Table 4. The most common type of e-cigarette used in the past 30 days was disposables (53.5%). The most common flavor of e-cigarette used in the past 30 days was fruit (48.8%). The most frequently reported source of e-cigarettes in the past 30 days was a friend (37.2%). Among students who reported buying the e-cigarettes themselves in the past 30 days, gas station/convenience stores and vape/tobacco shops were the most frequently reported sources. 

**Figure 1:**
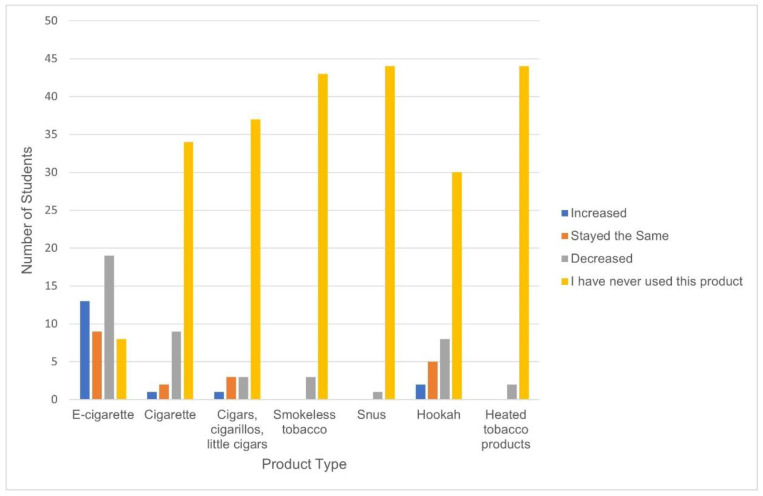
Product use behavior change due to the COVID-19 pandemic, NJYTS 2021 (n = 52).

Figure 1 illustrates the reported product use during the COVID-19 pandemic. Within each product category, a majority of product users reported decreased use during the pandemic.

## 4. Discussion

This study explored the feasibility of an online survey platform while examining student tobacco use and behavior patterns in a convenience sample of NJ high schools. Overall, 18.9% of our sample had ever used e-cigarettes and 6.1% currently used e-cigarettes. Although not directly comparable, our findings suggest a downward trend in e-cigarette use from 2018, when we found that 28.7% of students had ever used e-cigarettes and 17.8% of students reported the current use of e-cigarettes [6].

There was a notable increase in hookah use, though given the convenience sample, this finding should be interpreted with caution. This observed uptick could partially be explained by our disproportionately Hispanic sample, since the 2018 NJYTS found that ever use and current use of hookah were most prevalent among Hispanic students [6]. However, if genuine, this increase could be an unintended consequence of limited access to flavored e-cigarettes and warrants further study. All non-tobacco-flavored e-cigarettes (especially fruit-flavored) appear more popular than tobacco-flavored. Less than 10% of students who reported current e-cigarette use reported using tobacco-flavored e-cigarettes, which is of interest since flavors other than tobacco should not be available for purchase in New Jersey as of April 2020 [9]. 

The most commonly used device type was a disposable e-cigarette, which is consistent with findings from the 2021 National Youth Tobacco Survey [4]. Previous administrations reported that the most used device type in 2019 and 2020 was a prefilled pod or cartridge and that disposable e-cigarette use had increased significantly among youths who currently used e-cigarettes [10]. This shift in device type popularity correlates with increases in sales of disposable e-cigarettes nationally [11].

Another notable finding was the reported availability of e-cigarettes. All the students surveyed were under 21 years old and were able to access and purchase e-cigarettes, even though the age of sale has been 21 in New Jersey since November 2017 and nationally since December 2019 [12,13]. 

Future surveys should note whether the survey is completed in person in the classroom or remotely. The 2021 National Youth Tobacco Study was also fully conducted during the COVID-19 pandemic and found that youths who participated in a school building or classroom reported a higher prevalence of e-cigarette use (15%) than those who participated at home or in some other place (8%) [4,14]. In the current study, students reported an overall decreased use of tobacco products during the COVID-19 pandemic. This could be due to a similar phenomenon observed in the 2021 NYTS; namely, students may have been reluctant to report product use if they were taking the survey from home. Decreased use during the pandemic may have also been due to the lockdowns and inability to purchase products as well as decreased access to peers who could provide them. We anticipate that students will report increased use in the 2022 NJYTS because many COVID-19 restrictions may be relaxed at that time.

Our study was generally successful under difficult circumstances. Overall, the feedback from the schools was positive. Based on school feedback, future web-based school surveys should be mindful of school/district firewalls, simplify any class information forms, have a data collector on site if possible, and use individual sign-in cards to limit issues relating to multiple sign-ins. However, our results are consistent with previous findings that electronic survey administrations in schools are generally well-received and, importantly, can reduce time between data collection and dissemination [8,15,16]. 

The main limitation of this study was the small convenience sample of New Jersey high schools. This sample is not generalizable to all high schools in New Jersey; it disproportionately comprised Hispanic and Asian students relative to New Jersey high school enrollments. Another limitation of our study was the inability to control survey location (i.e., whether the student was at home or in school when they took the survey), which varied across schools and across classrooms within schools. Future administrations, even if carried out electronically, may be different outside of pandemic remote learning.

## 5. Conclusions

The 2021 NJYTS demonstrated the strengths and weaknesses associated with the use of an online platform. Future administrations will use this platform with our findings in mind. Given our small convenience sample, these data cannot be used to determine causality or policy implications. However, the reported student use of and access to flavored e-cigarettes provide evidence that such products remain accessible to youth. Despite a statewide ban on the sale of flavored e-cigarettes, young people face few barriers to finding such products. Continued monitoring of the access to and use of flavored products in young people is critical to evaluate the short- and long-term impacts of flavor policies. 

## Figures and Tables

**Table 1:** Sample demographics, NJYTS 2021 (n = 702).

	N	%	95% Confidence Interval	NJ High School Enrollment %
Gender				
Male	306	43.6	39.9–47.3	50.5
Female	356	50.7	47.0–54.4	49.5
Missing	40	5.7	4.0–7.4	
Race/Ethnicity				
White	149	21.2	18.2–24.3	44.6
Black	58	8.3	6.2–10.3	14.1
Hispanic	264	37.6	34.0–41.2	28.7
Asian	178	25.4	22.1–28.6	10.5
Other	9	1.3	0.5–2.1	2.2
Missing	44	6.3	4.5–8.1	
Grade				
9	144	20.5	17.6–23.5	25.4
10	236	33.6	30.1–37.1	25.1
11	176	25.1	21.9–28.3	24.9
12	107	15.2	12.6–17.9	24.6
Missing	39	5.6	3.9–7.3	

**Table 2:** Ever use of tobacco products, NJYTS 2021 (n = 702).

	Cigarette	Cigar	SLT	Hookah	Snus	E-cig	Heated	Nicotine Pouch
	N (%)	N (%)	N (%)	N (%)	N (%)	N (%)	N (%)	N (%)
Gender								
Male	15 (5.0)	18 (6.0)	6 (2.0)	16 (5.4)	1 (0.3)	46 (15.5)	1 (0.2)	2 (0.3)
Female	25 (7.1)	8 (2.3)	3 (0.9)	29 (8.3)	1 (0.3)	75 (21.6)	4 (0.7)	3 (0.5)
Race/Ethnicity								
White	8 (5.4)	12 (8.2)	5 (3.5)	7 (4.9)	1 (0.7)	37 (25.9)	1 (0.2)	2 (0.3)
Black	0 (0.0)	2 (3.5)	0 (0.0)	3 (5.3)	0 (0.0)	12 (21.4)	0 (0.0)	0 (0.0)
Hispanic	29 (11.0)	13 (5.0)	3 (1.2)	34 (13.0)	1 (0.4)	63 (24.2)	4 (0.7)	3 (0.5)
Asian	2 (1.1)	0 (0.0)	1 (0.5)	1 (0.6)	0 (0.0)	10 (5.5)	0 (0.0)	0 (0.0)
Other	0 (0.0)	0 (0.0)	0 (0.0)	1 (11.1)	0 (0.0)	0 (0.0)	0 (0.0)	0 (0.0)
Grade								
9	5 (3.6)	2 (1.4)	0 (0.0)	7 (5.0)	1 (0.7)	15 (10.7)	2 (0.3)	2 (0.3)
10	15 (6.4)	8 (3.5)	0 (0.0)	16 (6.9)	0 (0.0)	49 (21.2)	0 (0.0)	0 (0.0)
11	5 (2.9)	11 (6.4)	4 (2.3)	7 (4.1)	1 (0.6)	33 (19.4)	0 (0.0)	0 (0.0)
12	14 (13.2)	6 (5.7)	5 (4.7)	16 (15.1)	0 (0.0)	25 (23.8)	3 (0.5)	3 (0.5)
Overall	40 (6.1)	27 (4.1)	9 (1.4)	46 (7.1)	2 (0.3)	122 (18.9)	5 (0.8)	5 (0.8)

**Table 3:** Current (past 30 days) use of tobacco products, NJYTS 2021 (n = 702).

	Cigarette	Cigar	SLT	Hookah	Snus	E-cig	Heated	Nicotine Pouch
	N (%)	N (%)	N (%)	N (%)	N (%)	N (%)	N (%)	N (%)
Gender								
Male	2 (0.6)	6 (2.0)	1 (0.3)	3 (1.0)	0 (0.0)	18 (6.0)	0 (0.0)	0 (0.0)
Female	4 (1.1)	0 (0.0)	0 (0.0)	7 (2.0)	0 (0.0)	25 (7.1)	1 (0.2)	0 (0.0)
Race/Ethnicity								
White	2 (1.3)	4 (2.7)	1 (0.7)	0 (0.0)	0 (0.0)	11 (7.5)	0 (0.0)	0 (0.0)
Black	0 (0.0)	0 (0.0)	0 (0.0)	2 (3.5)	0 (0.0)	6 (10.5)	0 (0.0)	0 (0.0)
Hispanic	4 (1.5)	2 (0.8)	0 (0.0)	8 (3.0)	0 (0.0)	24 (9.1)	1 (0.2)	0 (0.0)
Asian	0 (0.0)	0 (0.0)	0 (0.0)	0 (0.0)	0 (0.0)	2 (1.1)	0 (0.0)	0 (0.0)
Other	0 (0.0)	0 (0.0)	0 (0.0)	0 (0.0)	0 (0.0)	0 (0.0)	0 (0.0)	0 (0.0)
Grade								
9	1 (0.7)	0 (0.0)	0 (0.0)	0 (0.0)	0 (0.0)	5 (3.5)	1 (0.2)	0 (0.0)
10	2 (0.9)	1 (0.4)	0 (0.0)	1 (0.4)	0 (0.0)	15 (6.4)	0 (0.0)	0 (0.0)
11	2 (1.1)	5 (2.9)	1 (0.6)	4 (2.3)	0 (0.0)	14 (8.1)	0 (0.0)	0 (0.0)
12	1 (0.9)	0 (0.0)	0 (0.0)	5 (4.7)	0 (0.0)	9 (8.4)	0 (0.0)	0 (0.0)
Overall	6 (0.9)	6 (0.9)	1 (0.1)	10 (1.4)	0 (0.0)	43 (6.1)	1 (0.1)	0 (0.0)

**Table 4:** E-cigarette use behaviors among students who used e-cigarettes in the past 30 days, NJYTS 2021 (n = 43).

	n (%)
Type of e-cigarette	
Disposable	23 (53.5)
Prefilled or refillable pods or cartridges	10 (23.3)
Tank	2 (4.7)
I don’t know the type	7 (16.3)
Flavor ^a^	
Mint or wintergreen	5 (11.6)
Menthol	10 (23.2)
Fruit	21 (48.8)
Alcohol or wine	2 (4.7)
Sweet, candy-like	14 (32.6)
Regular tobacco	4 (9.3)
Other	2 (4.7)
How students obtained e-cigarettes ^a^	
I bought them myself	13 (30.2)
I had someone else buy them for me	5 (11.6)
I asked someone to give me some	7 (16.3)
Someone offered them to me	10 (23.3)
I got them from a friend	16 (37.2)
I got them from a family member	2 (4.7)
I took them from a store or another person	3 (7.0)
I got them in some other way	1 (2.3)
Where e-cigarettes were purchased ^ab^	
I bought them from another person (a friend, family member, or someone else)	2 (15.4)
A gas station or convenience store	5 (38.5)
A grocery store	1 (7.7)
A drugstore	3 (23.1)
A mall or shopping center kiosk/stand	2 (15.4)
A vending machine	1 (7.7)
On the internet (such as a product website or store website like eBay or Facebook Marketplace), through the mail, or through a delivery service (such as DoorDash or Postmates)	1 (7.7)
A vape shop or tobacco shop	5 (38.5)
Some other place not listed here	0 (0.0)

^a^ Not mutually exclusive. ^b^ Among students who indicated they bought e-cigarettes themselves (n = 13).

## Data Availability

The data are not publicly available due to potential compromise of participant privacy.
